# Robust longitudinal multi-cohort results: The development of self-control during adolescence

**DOI:** 10.1016/j.dcn.2020.100817

**Published:** 2020-07-04

**Authors:** M.A.J. Zondervan-Zwijnenburg, J.S. Richards, S.T. Kevenaar, A.I. Becht, H.J.A. Hoijtink, A.J. Oldehinkel, S. Branje, W. Meeus, D.I. Boomsma

**Affiliations:** a Utrecht University, Utrecht, the Netherlands; b University of Groningen, University Medical Centre Groningen, Groningen, the Netherlands; c Vrije Universiteit Amsterdam, Amsterdam, the Netherlands; d Leiden University, Leiden the Netherlands

**Keywords:** Research synthesis, Informative hypotheses, Longitudinal analysis, Self-control, Sex differences

## Abstract

Longitudinal data from multiple cohorts may be analyzed by Bayesian research synthesis. Here, we illustrate this approach by investigating the development of self-control between age 13 and 19 and the role of sex therein in a multi-cohort, longitudinal design. Three Dutch cohorts supplied data: the Netherlands Twin Register (NTR; *N* = 21,079), Research on Adolescent Development and Relationships-Young (RADAR-Y; *N* = 497), and Tracking Adolescents’ Individual Lives Survey (TRAILS; *N* = 2229). Self-control was assessed by one measure in NTR and RADAR-Y, and three measures in TRAILS. In each cohort, we evaluated evidence for competing informative hypotheses regarding the development of self-control. Subsequently, we aggregated this evidence over cohorts and measures to arrive at a robust conclusion that was supported by all cohorts and measures. We found robust evidence for the hypothesis that on average self-control increases during adolescence (i.e., maturation) and that individuals with lower initial self-control often experience a steeper increase in self-control (i.e., a pattern of recovery). From self-report, boys have higher initial self-control levels at age 13 than girls, whereas parents report higher self-control for girls.

## 1 Introduction

It has become increasingly clear that researchers should replicate their work in different settings and conduct robustness checks to present informative and persuasive findings (Duncan et al., 2014). Coordinated multi-cohort analyses are important to establish the robustness of results (Duncan et al., 2014; Weston et al., 2019). A challenge in obtaining robust results for multi-cohort analyses is harmonization: how to synthesize data that assess the same concept but have been based on varying questions or subsets of items (Hofer and Piccinin, 2009). Multi-cohort efforts can be combined at the level of the data (e.g., integrative data analysis; IDA; Curran et al., 2008), the parameters (e.g., fixed or random effects meta-analysis), or the hypotheses (Kuiper et al., 2012). A drawback of IDA and meta-analysis is that these approaches yield average results instead of findings that are robust across studies, while robustness is of importance to research and its generalization. As we aim to show in the current study, Bayesian research synthesis enables researchers to examine robustness of effects across different measures of the same concept and across cohorts.

Consider the case of self-control: very briefly, self-control is a process to inhibit inappropriate dominant impulses and responses in favor of appropriate ones (Casey, 2015; Nigg, 2017; Willems et al., 2018). Self-control covers the top-down aspect of behavioral control, i.e., it is an effortful or executive mechanism as opposed to reactive or responsive mechanisms like fear and inhibition. Cortical structures, the anterior cingulate cortex (ACC) and the dorsolateral, ventrolateral and ventromedial prefrontal cortex serve the self-control process (Bridgett et al., 2015; Nigg, 2017). Self-control can be measured by scales from over a hundred self-control and personality questionnaires (Duckworth and Kern, 2011). In Bayesian research synthesis, support is evaluated for competing hypotheses that should apply to all measurement methods in the study. Researchers who are interested in self-control generally do not hypothesize diverging results for different self-control questionnaires; that would imply that the focus is not on self-control as such, but on ‘self-control scores on questionnaire X’. In other words, if different measures are valid and are expected to evaluate the same concept, similar findings are anticipated for each of them.

The competing hypotheses in Bayesian research synthesis are informative hypotheses (Hoijtink, 2012) about the parameters in the model. In the present study, we will use growth curve models in which each subject’s development of self-control is estimated by an intercept (the initial level) and a slope (the development). Whereas a classical null hypothesis states that the parameter of interest is equal to zero (e.g., H_0_: the mean of the individual slopes, ɑ_S,_ = 0), informative hypotheses can also include range constraints (e.g., ɑ_S_ > 0; ɑ_S_ > 0.20; 0.20 < ɑ_S_ < 0.50; etc.), orderings of parameters (e.g., ɑ_Sgroup1_ > ɑ_Sgroup2_ > ɑ_Sgroup3_), or combinations of these (e.g., ɑ_S group3_ > 0.20 & ɑ_Sgroup1_ > ɑ_Sgroup2_ > ɑ_Sgroup3_; ɑ_Sgroup1_ - ɑ_Sgroup2_ > 0.20, etc.). After the set of competing hypotheses has been specified, the evidence for each hypothesis is evaluated for each cohort and measure separately. The relative support for each of the hypotheses in the set is expressed in posterior model probabilities (PMPs), which add up to 1.00. Subsequently, the PMPs can be aggregated over measures and cohorts. The result of the aggregation is the relative support for each hypothesis in the set by all cohorts and assessment methods simultaneously. The best supported hypothesis is robustly supported, irrespective of cohort specific characteristics and measurement materials.

Zondervan-Zwijnenburg et al. (2019) and Veldkamp et al. (2020, in press) applied Bayesian research synthesis to cross-sectional data from multiple cohorts on the association of parental age and offspring behavioral problems as assessed with different instruments. Here we demonstrate how Bayesian research synthesis can also be applied in multi-cohort longitudinal analyses. It is essential for the progress of developmental sciences, that research findings are accumulated over independent longitudinal studies (Hofer and Piccinin, 2009; Butz and Torrey, 2006). While multi-cohort cross-sectional analyses are mainly challenged by diverging measurement instruments, longitudinal analyses also bring within-study differences in items over time and between-study differences in the timing of assessments. These challenges sometimes obstruct planned meta-analyses (see, for example, Park et al., 2003) or integrative data analyses (see, for example, Hussong et al., 2008).

In this paper, we applied Bayesian research synthesis on a multi-outcome and multi-cohort longitudinal analysis of adolescent self-control. Specifically, we first investigated (1) typical self-control development patterns across adolescence (ages 13–19 years), and (2) the relation between self-control levels centered at age 13 and further self-control development. As a follow-up, we investigated potential sex differences in the development of self-control. The literature on self-control that led to the competing informative hypotheses evaluated in the Bayesian research synthesis procedure is discussed in Section 2.5.1

## 2 Materials & methods

All data-preparation and analysis scripts can be found at osf.io/r2tyk. Simulated data that can be used to run the scripts is also provided.

### 2.1 Participants

The three cohort studies that contributed to the current study were the Netherlands Twin Register (NTR; Bartels et al., 2007; Ligthart et al., 2019), the Research on Adolescent Development and Relationships-Young cohort (RADAR-Y; Branje and Meeus, 2018), and the Tracking Adolescents’ Individual Lives Survey (TRAILS; Oldehinkel et al., 2015). The cohorts provided data for participants between 10 and 24 years old with at least one self-control assessment. Parental consent and child assent were obtained for all minors. Data from all ages were used to handle missing data with multiple imputation, but the final analyses only included data from participants between the ages of 13 and 19 years old, as this age range was covered with self-control assessments in all three cohorts. The descriptive statistics in this paper concern this group of participants per cohort.

The NTR sample consisted of 21,079 participants of whom 42.8 % were male. They were twins, triplets, or siblings of twins. Mother’s education was low (i.e., elementary education) for 3.7 %, medium (i.e., secondary education, vocational training) for 70.1 %, and high (i.e., university) for 26.2 %. Most participants were of Dutch origin (93.9 %). The RADAR-Y sample consisted of 497 participants, of whom 56.9 % was male. Mother’s education was low for 3.2 %, medium for 56.6 %, and high for 40.2 %. Parents of 92.1 % of the participants were born in the Netherlands. The TRAILS sample consisted of 2229 participants, of whom 49.3 % were male. Mother’s education was low for 6.8 %, medium for 66.4 %, and high for 26.8 % of the cohort. Most participants were of Dutch origin (86.5 %).

### 2.2 Measures

#### 2.2.1 Self-Control

Self-control was defined as the ability to inhibit inappropriate dominant impulses and responses in favor of appropriate ones (Casey, 2015; Nigg, 2017; Willems et al., 2018). One measure for self-control is the ASEBA Self-Control scale (ASCS; Willems et al., 2018, see items in Table 1). In ASEBA questionnaires (i.e., Child Behavior Checklist, CBCL; Youth Self-Report, YSR; Young Adult Self-Report, YASR, Adult Self-Report, ASR; Achenbach et al., 2017), self-control problems are rated at a three-point scale with the answering options: 0 = *not true*, 1= *somewhat or sometimes true*, and 2 = *very true or often true*. The 8-item ASCS instrument was repeatedly assessed in NTR (after age 12/13 self-reported), TRAILS (child-reported at waves 1–4 and parent-reported at waves 1–3), and partly in RADAR-Y (child-reported at waves 2–7). The ASCS items were recoded such that higher scores reflect more self-control. In RADAR-Y the aggression and rule-breaking items of the ASCS were included, but not the items covering attention problems. RADAR-Y participants completed the Difficulties in Emotion Regulation Scale (DERS; Gratz & Roemer, 2004), which includes a *Difficulties in Goal-Directed Behavior* scale with items on getting work done and focusing when being upset (see items in Table 1). The answering categories range from 1 = *almost never* to 5 = *almost always*. The ASCS aggression and rule-breaking items in combination with the DERS *Difficulties in Goal-Directed Behavior* scale together cover the concept of self-control and closely match the assessment by the full ASCS. Also, for the DERS, items were recoded into positive assessments of self-control.

**Table 1:** Questionnaires and Items to Measure Self-Control.

NTR	TRAILS	RADAR-Y
*ASCS Self-reported*	*ASCS Self-reported / Parent-reported*	*ASCS-DERS*
Break rules at home, school, or elsewhere	Break(s) rules at home, school, or elsewhere	Breaks rules at home, school, or elsewhere
Stubborn, sullen, or irritable	Stubborn, sullen, or irritable	Stubborn, sullen, or irritable
Sudden changes in mood or feelings	Sudden changes in mood or feelings	Sudden changes in mood or feelings
Temper tantrums or hot temper	Temper tantrums or hot temper	Temper tantrums or hot temper
Impulsive or act without thinking	Impulsive or act(s) without thinking	–
Fail to finish what I start	Fail(s) to finish what I start / he/she starts	–
Can’t concentrate, can’t pay attention for long	Can’t concentrate, can’t pay attention for long	–
Inattentive or easily distracted	Inattentive or easily distracted	–
		When I’m upset, I have difficulty getting work done
		When I’m upset, I have difficulty concentrating
		When I’m upset, I have difficulty focusing on other things
		When I’m upset, I have difficulty thinking about anything else
	*EATQ Parent-reported*	
	Follows plan to finish projects (R)	
	Easy to concentrate on homework problems (R)	
	Hard to ignore background noises	
	Pay close attention to verbal instructions (R)	
	When interrupted, forgets what saying	
	Can keep track of different things (R)	

*Note.* (R) indicates that an item is reverse-coded.

For the TRAILS participants, one of the parents (usually the mother) also responded to items of the Early Adolescence Temperament Questionnaire Revised (EATQ-R; Ellis & Rothbart, 2001) at waves 1, 3 and 4. We included the items of the *Attention Control* and *Inhibitory Control* scale that were repeatedly assessed (see items in Table 1). The *Attention Control* scale of the EATQ-R assesses the ability to focus and sustain attention as well as to shift attention when desired. The *Inhibitory Control* scale assesses the ability to suppress or stop inappropriate behaviors, wait and plan before acting. Answering categories range from 1= *almost always untrue* to 5 = *almost always true*. Some EATQ-R items were recoded such that higher scores reflect more self-control.

In sum, self-control was measured with the self-reported ASCS in NTR and TRAILS, the self-reported ASCS-DERS combination in RADAR-Y, and the parent-reported ASCS and EATQ-R in TRAILS. Whereas the ASCS measures self-control problems, the DERS and EATQ-R cover a completer spectrum from low to high self-control. Table 1 gives an overview of all items per measure. Table 2 shows how many observations were present at each age and the total number of observations. Table 3 gives the number of assessments per person. Figure S1, S2 and S3 present how assessments are distributed over ages for NTR, RADAR-Y and TRAILS respectively. These tables and figures show a preview of Sections 2.3.1 and 2.3.2 in which within- and between-study differences are discussed in more detail.

**Table 2:** Number of Observations by Age per Self-Control Measure.

Age	13	14	15	16	17	18	19	Total
NTR ASCS	727	5074	4796	4549	5679	4722	3508	29,055
TRAILS ASCS	957	1162	304	1319	492	632	1194	6060
TRAILS P-ASCS	957	1162	304	1319	492	194	1	4429
TRAILS EATQ	7	0	223	1319	492	632	1194	3867
RADAR-Y ASCS-DERS	46	435	494	496	496	452	172	2591

*Note.* ASCS = ASEBA Self-Control scale, P-ASCS = Parent-reported ASCS, EATQ = Early Adolescence Temperament Questionnaire Revised.

**Table 3:** Number of Participants by Number of Assessments per Self-Control Measure.

Number of Assessments	1	2	3	4	5	6
NTR ASCS	14,310	5575	1181	13		
TRAILS ASCS		627	1602			
TRAILS P-ASCS	36	2186	7			
TRAILS EATQ	591	1638				
RADAR-Y ASCS-DERS			1	2	384	110

*Note.* ASCS = ASEBA Self-Control scale, P-ASCS = Parent-reported ASCS, EATQ = Early Adolescence Temperament Questionnaire Revised.

#### 2.2.2 Covariates

Sex was included as a covariate and recoded such that in each cohort boys were the reference category (i.e., 0) and girls were coded 1.

### 2.3 Data structure

Challenges in research synthesis for longitudinal studies are within-study differences in items over time and between-study differences in the timing of assessments. We explain how we dealt with these issues below.

#### 2.3.1 Within-study differences in items

The NTR study followed multiple cohorts of twins since 1987, with different questionnaires for different age groups; also, some questionnaires have been updated over time. NTR included three ASEBA self-report instruments: the Young Adult Self-Report (YASR), Youth Self-Report (YSR) and the Adult Self-Report (ASR). The YASR, which was part of five assessments, did not include the “*inattentive or easily distracted*” item of the ASCS and the items “*failing to finish*” and “*breaking rules*” were not included in two out of five YASR assessments. The “*inattentive or easily distracted*” item is not covered in the Adult Self-Report (ASR), which was administered twice with older adolescents and young adults. The YSR, which includes all ASCS items was assessed in a subgroup of older adolescents of with the same age as those that filled out the YASR and ASR (see also Table 4 and Supplementary Figure S1). Thus, missing data for participants who lacked specific items could be imputed with multiple imputation software using the information from participants with the same age with information on all items.

**Table 4:** Questionnaire Versions by Age per Measure (see also 2.3.1).

*Note.* ASCS = ASEBA Self-Control scale, P-ASCS = Parent-reported ASCS, EATQ = Early Adolescence Temperament Questionnaire Revised.

In RADAR-Y, the DERS scale was assessed at Waves 2–7 (see also Figure S2). Consequently, we only had DERS data for participants in the age range 13–19. We decided to take the age-range covered by the DERS scale (i.e., 13–19) as the age-range for our study.

In TRAILS, the YSR was assessed at Wave 3, while the ASR was assessed at Waves 4 and 5 when participants were older than 18 years (see Figure S3). Hence, there were no Wave 4 and 5 data on the *inattention and distraction* item at all. As 151 18- and 19-year-old participants filled in the *inattention and distraction* item in Wave 3, scores from these participants were used to impute this item for 18- and 19-year-olds in Wave 4. The same issue was resolved likewise for two EATQ items: “*If my child is distracted or disturbed, (s)he forgets what (s)he was saying*” and “*My child finds it hard to ignore background noises to concentrate on schoolwork*”. Another within-study difference in TRAILS was that the EATQ was not assessed at Wave 2, which meant few EATQ data for 13-year-olds and no EATQ data for 14-year-olds. This within-study difference could not be tackled with imputation strategies. Hence, the EATQ analysis has data from 15-year-olds only.

In short, changing sets of items over assessments within cohorts were approached as a missing data problem and could be resolved by rearranging data by age and applying multiple imputation. If a questionnaire was missing for a whole wave and age group, these data could not be imputed, and the missing age group could not be included in the analysis.

#### 2.3.2 Between-study differences in timing of assessments

The three cohort studies were all characterized by a longitudinal design, but with different sampling strategies and assessment intervals.

RADAR-Y and TRAILS both followed a pre-selected cohort over time. In RADAR-Y, the cohort was assessed almost yearly. Figure S2 shows the distribution of age over waves 1–9, of which Waves 2–7 were included in our study. The TRAILS cohort had assessments about every 2.8 years of which four waves (wave 1–4) with ASCS self-reports could be included. See Figure S3 for the distribution of age over Waves 1–5. Three parent-reported ASCS assessments (not included in Wave 4) and three parent-reported EATQ assessments (not included in Wave 2) were available in the same age range.

NTR data for 12 to 24-year-old participants came from two sources. The first one is the Young NTR cohort in which twins have been recruited since 1987, typically shortly after birth with their siblings joining at later ages (Lamb et al., 2010). Twins and their siblings received self-report surveys at ages 12, 14, 16 and 18 years. A subgroup first received a pilot assessment of these surveys. The second data source was the Adult NTR cohort, which began in 1991 by recruiting adolescent and young adult twins and family members (Boomsma et al., 2002) through city councils. The YASR / ASR were included in ANTR surveys 1 (1991), 3, 4, 5, 8 and 10 (around 2013). YNTR participants who reached age 18 years could participate in ANTR surveys 8 and / or 10. In addition, a survey including the ASR is sent to new adult participants. Over both NTR data sources, a total of 12 assessments (4 YNTR + 1 pilot, 6 ANTR + 1 ANTR new participants) were available from 12 to 24-year-old participants (see Supplementary Figure S1 for the distribution of age over assessments).

To run comparable longitudinal analyses between the cohort studies, the final data structure needed to be by participants’ age in years instead of by wave or assessment. After applying multiple imputation on the items (see Van Buuren, 2018 and Supplementary Material for details), self-control sum scores per age 13–19 were constructed. If a participant did not participate in an assessment at a certain age, data were not imputed for that age.

### 2.4 Analyses

**Fig. 1:**
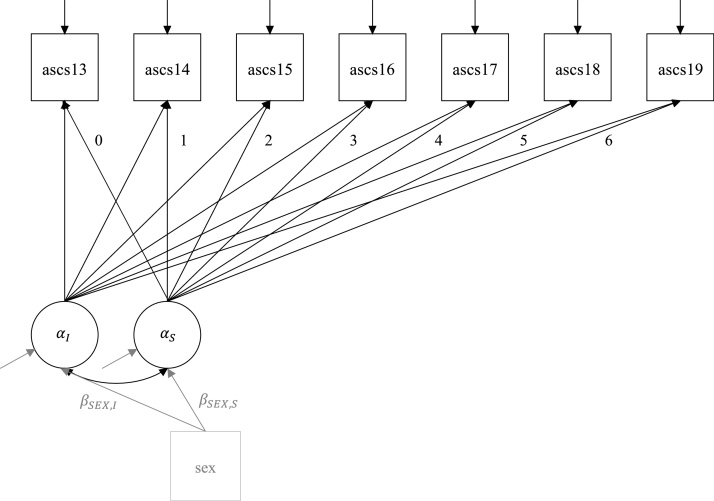
Statistical models. Model 1 in black: Latent growth model with repeated measures by age from 13 to 19 years on top, depicted for the dependent variable ASCS. The values 0-6 are the factor loadings for the slope factor. ɑ_I_ and ɑ_S_ are the means of the latent growth intercept and slope respectively, and σ_I,S_ is the covariance between the intercept and slope latent growth factors. Model 2 = Model 1 + sex as a predictor of the intercept and slope factor with coefficients of interest: β_SEX,I_ and β_SEX,S_.

The first analysis was a latent growth model with an intercept and slope (see Fig. 1, in black). The intercept was set at the first included assessment at age 13, where the data was also centered. In this model we evaluated the linear development of self-control (i.e., the mean of the slope, ɑ_S_) and the relation between initial levels of self-control and its development (i.e., the covariance between the intercept and slope, σ_I,S_). Although interesting, we could not model quadratic effects for each cohort, due to the limited number of repeated observations per person (see Table 3). The latent growth model was fitted to the data for the 3 cohorts separately. In TRAILS, a multivariate latent growth model with correlated intercepts and slopes was constructed in Mplus 8.4 (Muthen & Muthen, 1998-2017), to take covariances between the growth factors for the three measures of self-control into account. In the second model, sex was included as a predictor of the intercept and slope (see Fig. 1, in grey). Again, this analysis was conducted for each cohort separately. For NTR, all analyses were executed with a cluster-correction on family ID, to obtain correct standard errors. The runMI function of the SEMtools R-package (Jorgensen et al., 2019) was used to obtain lavaan (Rosseel, 2012) results that were pooled over imputations.

### 2.5 Bayesian research synthesis

The core concept of Bayesian research synthesis was introduced by Kuiper et al. (2012) and elaborated upon by Zondervan-Zwijnenburg et al. (2019). In Sections 2.5.1−2.5.3 we explain the steps for evaluating the development of self-control: constructing informative hypotheses, obtaining PMPs and applying Bayesian research synthesis.

#### 2.5.1 Constructing informative hypotheses

We based our informative hypotheses on the literature (see also elsewhere in this special issue) and only briefly discuss some main findings with respect to the development of self-control in adolescence that led to our set of informative hypotheses.

Longitudinal studies on self-control levels from early to late adolescence have mostly reported decreasing problems over age, suggesting maturation (Burt et al., 2014; Casey, 2015; Shulman et al., 2015). These findings are consistent with prominent theories that predict increase of cognitive control across adolescence: the Dual Systems model (Steinberg et al., 2008) and the Maturational Imbalance model (Casey, Getz & Galvan, 2008). However, large groups of adolescents showing stability were also observed (Khurana et al., 2018). Given this literature, we expected that the mean of the linear slope of self-control would be either > 0 or 0, meaning that self-control increases or is stable over age. With respect to the association between initial levels of self-control and further development, we hypothesized about the absence of a relation (i.e., σ_I,S_ = 0), recovery (i.e., σ_I,S_ < 0), or progressive decline (i.e., σ_I,S_ > 0). Recovery means that higher initial self-control is related to a lower increase in self-control. Progressive decline means that higher initial levels of self-control are related to more increase in self-control over age. Thus, for the latent growth model without predictors, we considered the following competing hypotheses:

H1 ɑ_S_ = 0, σ_I,S_ = 0, on average self-control is stable, and there is no evidence for progressive decline or recovery.

H2 ɑ_S_ = 0, σ_I,S_ > 0, on average self-control is stable, and there is variance among participants and evidence for progressive decline.

H3 ɑ_S_ = 0, σ_I,S_ < 0, on average self-control is stable, and there is variance among participants and evidence for recovery.

H4 ɑ_S_ > 0, σ_I,S_ = 0, on average there is self-control maturation and there is no evidence for progressive decline or recovery.

H5 ɑ_S_ > 0, σ_I,S_ > 0, on average there is self-control maturation, and there is variance among the participants and evidence for progressive decline.

H6 ɑ_S_ > 0, σ_I,S_ < 0, on average there is self-control maturation, and there is variance among the participants and evidence for recovery.

Ha ɑ_S_ < 0, σ_I,S_. Anything not captured in H_1_-H_6_.

In this set, H_a_ is the alternative hypothesis stating that ɑ_S_ is negative and σ_I,S_ can take on any value. This alternative hypothesis functions as a fail-safe, because it will receive most support if the other hypotheses do not represent the data well.

For model 2, the parameters of interest were the coefficients of sex predicting the latent growth factors in model 1 (i.e., β_SEX,I_, and β_SEX,S_). The general observation is that girls have more self-control than boys (i.e., β_SEX,I_ > 0; Chapple, Vaske & Hope, 2010, Shulman et al., 2015). However, this difference is not observed in every study (i.e., β_SEX,I_ = 0; e.g., Jonason & Tost, 2010). There is little evidence on sex-specific development of self-control over adolescence. From Turner and Piquero (2002), we can derive evidence for either a stable or an increasing difference between boys and girls over time (i.e, β_SEX,S_ = 0 or β_SEX,S_ > 0 respectively). Because recovery is an option in the previous model, we also considered the option that the difference between boys and girls decreases with age (i.e., β_SEX,S_ < 0).

The final set of hypotheses concerned every combination of the two coefficients with the intercept-regression being either equal to zero or positive (i.e., girls show equal or higher self-control) and all options open for the slope-regressions (i.e., negative, zero, or positive), resulting in six informative hypotheses. That is:

H1 β_SEX,I_ = 0, β_SEX,S_ = 0, on average, self-control at 13 and its development thereafter is equal for boys and girls

H2 β_SEX,I_ = 0, β_SEX,S_ < 0, on average, self-control at 13 is equal for boys and girls, but boys show less maturation over time compared to girls

H3 β_SEX,I_ = 0, β_SEX,S_ > 0, on average, self-control at 13 is equal for boys and girls, but boys show more maturation over time compared to girls

H4 β_SEX,I_ < 0, β_SEX,S_ = 0, on average, girls have more self-control at age 13 and this difference between boys and girls is stable over time.

H5 β_SEX,I_ < 0, β_SEX,S_ < 0, on average, girls have more self-control at age 13, and this difference increases over time.

H6 β_SEX,I_ < 0, β_SEX,S_ > 0, on average, girls have more self-control at age 13, but this difference decreases over time.

Ha β_SEX,I_ > 0, β_SEX,S_. Anything not captured in H_1_-H_6_.

#### 2.5.2 Obtaining posterior model probabilities

As a next step, the relative evidence for all hypotheses versus an alternative ‘anything can be true’ hypothesis was evaluated in each dataset with Bayes factors through the R-package bain (Gu et al., 2019) in R (R Core Team, 2019). The results were communicated with PMPs that cover the relative probability of each hypothesis within the set of evaluated hypotheses, summing up to 1.0. The hypothesis that received most support was considered the best hypothesis for that dataset. If the difference between the PMPs for the two best hypotheses is <.10, the hypotheses are considered to have a shared first position. Note that Bayes factors and their corresponding PMPs are related to sample size. Larger sample sizes increase estimate precision (i.e., smaller standard errors), leading to more pronounced evidence for or against the hypothesis of interest versus H_a_, as evaluated in the Bayes factor. Accordingly, the PMPs in a set also become more distinct with increasing sample sizes.

#### 2.5.3 Applying Bayesian research synthesis

Finally, aggregated PMPs were calculated for each hypothesis. Aggregated PMPs take the PMP of the previous cohort as a prior model probability for the current cohort’s PMP, until all cohorts have been taken into account. To compute PMPs for the first cohort, PMPs from a previous cohort are not available and we need to specify prior model probabilities by ourselves. We used equal prior model probabilities for all hypotheses, that is: $\pi^{0}=1/7$. Technically, the order of aggregating the cohorts and measures is not important, which means that with equal initial prior model probabilities, we can also take the product of the five PMPs (one for each instrument) for one hypothesis and divide it by the sum of the PMP products for each hypothesis (Kuiper et al., 2012) (i.e., $\frac{\prod_{v=1}^{V}\pi_{v,h}^{1}}{\sum_{h=1}^{H}\prod_{v=1}^{V}\pi_{v,h}^{1}}=\pi_{V,h}^{1},$ where *v* is variable 1, …, *V* = 5; *h* is hypothesis 1, …, *H* = 7; and $\pi^{1}$ is the PMP).

Aggregated PMPs indicate how much each hypothesis is supported by all datasets simultaneously. In essence it means that every aforemented hypothesis ended with “… in NTR, RADAR-Y and the three TRAILS questionnaires”. For example, H1 for model 1 becomes: H1: ɑ_S_ = 0, σ_I,S_ = 0 in NTR, RADAR-Y and the three TRAILS questionnaires. The end result was thus a set of probabilities (one for each hypothesis) that communicates how well each of the hypotheses was supported by all outcomes, irrespective of the population and measurement specifics. In other words, the result encompasses the robust support for each of the hypotheses of interest.

## 3 Results

Table 5 shows the results of the analysis of Model 1 with the probabilities rounded at two decimals. Please note that .00 means that the evidence is <.005, but not strictly 0. H_1_, H_3_, and H_6_ all received more than .70 probability in at least one evaluation. Hypotheses H_2_, H_5_, and H_a_ received very little support from all cohorts and operationalizations of self-control. Thus, we find that the probability of a positive covariance between the intercept and slope (i.e., progressive decline as captured in H_2_ and H_5_) is near zero, as is a negative slope for self-control (as captured in H_a_).

**Table 5:** Posterior Model Probabilities for the hypotheses concerning self-control development and its covariance with initial self-control levels.

	H1	H2	H3	H4	H5	H6	Ha
**NTR: ASCS**	.00	.00	.00	.06	.00	.*94*	.00
**RADAR-Y: ASCS-DERS**	.09	.00	.*81*	.00	.00	.03	.07
**TRAILS: ASCS**	.00	.00	.04	.00	.00	.*96*	.00
**TRAILS: Parent-ASCS**	.17	.00	.07	.*52*	.01	.24	.00
**TRAILS: EATQ**	.*72*	.02	.06	.18	.00	.02	.00
**All**	.00	.00	.00	.00	.00	**1.00**	.00

*Note.* Hypotheses: H1: ɑ_LS_ = 0 & σ_I,LS_ = 0, H2: ɑ_LS_ = 0 & σ_I,LS_ > 0, H3: ɑ_LS_ = 0 & σ_I,LS_ < 0, H4: ɑ_LS_ > 0 & σ_I,LS_ = 0, H5: ɑ_LS_ > 0 & σ_I,LS_ > 0, H6: ɑ_LS_ > 0 & σ_I,LS_ < 0, Ha: ɑ_LS_ < 0, σ_I,LS_.

**Fig. 2:**
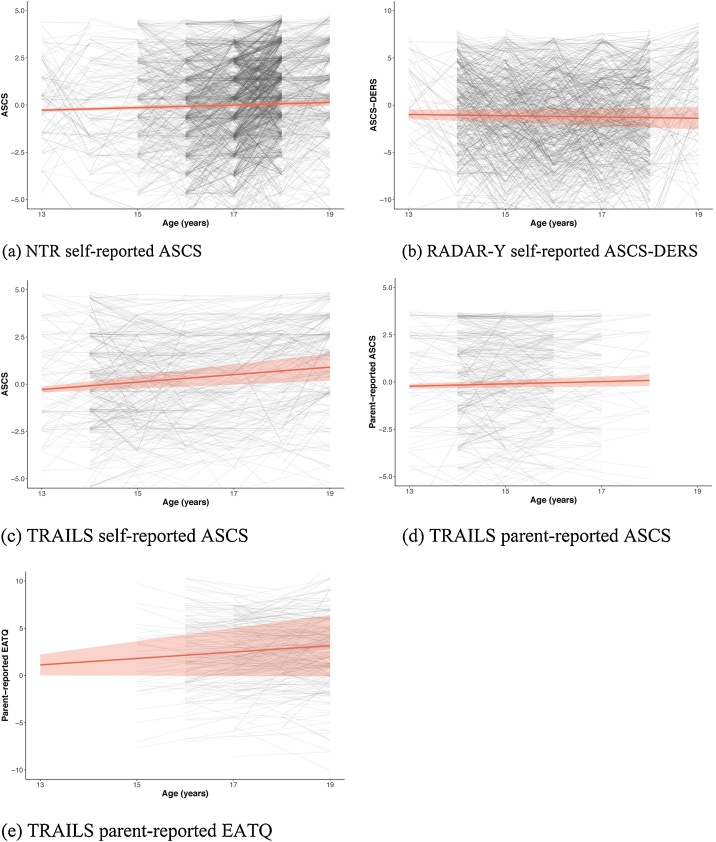
Development of self-control ±1*SE* for cohorts and measurement instruments.

When we look at the aggregated level with aggregated hypotheses (i.e., the aforementioned hypotheses followed by “… in NTR, RADAR-Y and the three TRAILS questionnaires”), the best supported hypothesis with a probability of 1.00 is H_6_: ɑ_LS_ > 0, σ_I,LS_ < 0 in NTR, RADAR-Y and the three TRAILS questionnaires; on average there is an increase in self-control, but there is variance among the participants with higher initial self-control going together with a lower increase in self-control (the negative covariance is also covered in H_3_). Arranged by strength, the slope effect sizes (i.e., slope divided by its standard deviation; Muthén & Muthén, 2002) per outcome were -0.09 (RADAR-Y), 0.17 (TRAILS P-ASCS), 0.25 (NTR), 0.59 (TRAILS ASCS), and 0.67 (TRAILS EATQ). The correlation between intercept and slope was -0.62 (TRAILS EATQ), -0.53 (NTR), -0.52 (TRAILS ASCS), -0.47 (RADAR-Y), -0.38 (TRAILS P-ASCS). Fig. 2 shows the predicted growth patterns (with standard error) for the different cohorts and instruments in red. On the background within-participant observations are connected with solid lines connecting consequetive ages, and dotted lines connecting non-consequetive ages.

In H_1_ and H_4_, the covariance between the slope and intercept at age 13 is zero. TRAILS Parent-ASCS and TRAILS EATQ support this, but the finding is not robust over all cohorts. A sensitivity analysis showed that when we evaluate the covariance between the linear slope and intercept at age 16, H_4_: ɑ_LS_ > 0 & σ_I,LS_ = 0 becomes the most plausible hypothesis (Table S1). Thus, the presence of recovery with regard to self-control may vary with age.

**Fig. 3:**
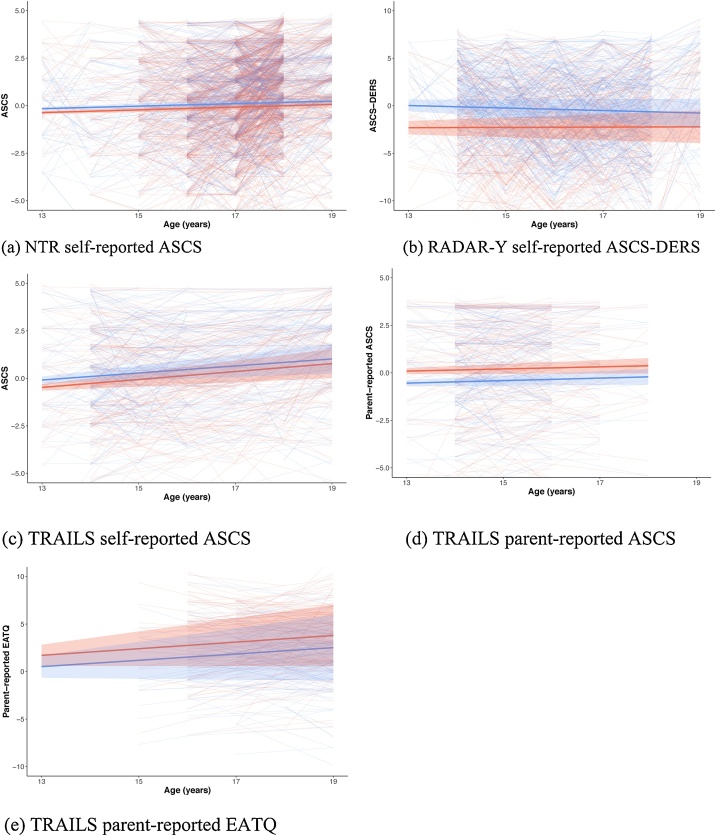
Development of self-control ±1*SE* for girls and boys for different cohorts and measurement instruments.

Table 6 shows the result for our analysis of Model 2, which included sex as a predictor of the intercept and slope. H_3_, H_4_, and H_a_ all received substantial support in at least one evaluation. With probabilities of .51 and .49 respectively, the best supported aggregated hypotheses are H_4_: β_SEX,I_ > 0 & β_SEX,S_ = 0 in NTR, RADAR-Y and the three TRAILS questionnaires; and H_a_: β_SEX,I_ < 0, β_SEX,S_ in NTR, RADAR-Y and the three TRAILS questionnaires. The effect sizes for the impact of sex (girls = 1) on the intercept were: -0.60 (RADAR-Y), -0.19 (TRAILS ASCS), -0.09 (NTR), 0.23 (TRAILS P-ASCS), and 0.34 (TRAILS EATQ). In H_a_, nothing was specified concerning β_SEX,S_. Notably, support for H_4_ comes from parent-reports, whereas support for H_a_ comes from self-report measures. Fig. 3 shows the predicted growth patterns (with a standard error) in red for girls and blue for boys. On the background within-participant observations are shown for girls and boys.

**Table 6:** Posterior Model Probabilities for the hypotheses concerning sex predicting the intercept and slope of self-control.

	H1	H2	H3	H4	H5	H6	Ha
**NTR: ASCS**	.05	.00	.*68*	.00	.00	.00	.26
**RADAR-Y: ASCS-DERS**	.00	.00	.00	.00	.00	.00	*1.00*
**TRAILS: ASCS**	.16	.00	.04	.00	.00	.00	.*80*
**TRAILS: Parent-ASCS**	.00	.00	.00	.*93*	.04	.03	.00
**TRAILS: EATQ**	.00	.01	.00	.*86*	.11	.02	.00
**All**	.00	.00	.00	.**51**	.00	.00	**.49**

*Note.* Hypotheses: H1: β_SEX,I_ = 0 & β_SEX,S_ = 0, H2: β_SEX,I_ = 0 & β_SEX,S_ > 0, H3: β_SEX,I_ = 0 & β_SEX,S_ < 0, H4: β_SEX,I_ > 0 & β_SEX,S_ = 0, H5: β_SEX,I_ > 0 & β_SEX,S_ > 0, H6: β_SEX,I_ > 0 & β_SEX,S_ <0, Ha: β_SEX,I_ < 0, β_SEX,S_.

## 4 Discussion

One of the challenges for social science is the accumulation of longitudinal data (Butz and Torrey, 2006). We showed that robust evidence over multiple measurement instruments and cohorts can be obtained by means of Bayesian research synthesis. Behind the robust overall results, the preferred hypothesis varied over cohorts and instruments. This advocates our robust approach: if one or two of the included studies separately published their results, we might have drawn different conclusions than from the synthesized results. Also, we did not observe structural similarities and differences between cohorts and measures. That is, the set of ASCS self-reports (NTR and TRAILS), the set of TRAILS outcomes, or the set of parent-reports did not prefer the same hypothesis with respect to the development of self-control. However, in the model with sex predicting the self-control intercept and slope, the parent-reports could be distinguished in their preference for H_4_. The distinction between self- and parent-reports could mean that parents and youth report differently on self-control, depending on the sex of the adolescent. Kevenaar et al. (2020 this special issue) show that rater effects are present for self-control. To establish the cause for these differences, our study with three cohorts and four different measures of self-control is a starting point. A study with a larger number of cohorts and questionnaires would be needed to test for systematic differences between cohorts or reports. As there are rater-effects, we may wonder if it is best to aggregate the parent- and self-reported results in one robust analysis, or whether data from different raters should be aggreagated separately and possibly one rater should be preferred over the other.

We also found that some hypotheses structurally received little to no support. In Model 1, three hypotheses (uniquely covering progressive decline, and increasing in self-control over age) received less than 5% relative probability from each cohort. In Model 2, three hypotheses received less than 10 % relative probability from each cohort. This means that based on our multi-cohort and multi-measure investigation, we can exclude those hypotheses from future research.

In line with most earlier theories and studies (Burt et al., 2014; Casey, 2015; Shulman et al., 2015; Steinberg et al., 2008), we found robust evidence for an increase in self-control throughout adolescence accompanied by a pattern of recovery (i.e., those with lower initial self-control levels experience more increase thereafter). We also found that variance around the average pattern was partly explained by sex, but the direction of the effect differed between self- and parent-reports. Opposite to our informative hypotheses, the robust support from self-reports prefferred the hypothesis in which boys show higher self-control than girls at age 13. Future research may explore whether this finding reflects rater differences, or whether biological differences between boys and girls play a role. Other factors explaining self-control levels and development involve cognition and educational levels and genetic variation (Willems et al., 2018). A limitation in our study is that raters reported on behavior resulting from an interplay between top-down and bottom-up processes, and not on the self-control process itself. Future research can also explore whether self-control problems develop in a quadratic fashion during adolescence. The observed data in Fig. 2 seem to imply that a quadratic effect may be present, but the number of repeated observations per person in most of our datasets was insufficient to model and evaluate such an effect. Building on the (robust) results of the current study, future research could also evaluate specific hypotheses, such as competing hypotheses on specific effect sizes for self-control development.

### 4.1 Conclusion

We applied Bayesian research synthesis to evaluate the development of self-control problems during adolescence and its prediction by sex. With this method, we found robust evidence for the hypothesis that self-control generally increases in adolescence and that youth with more higher self-control have a lower increase in self-control over age. Thus, we see a pattern of maturation and recovery. Furthermore, we found that boys report higher self-control levels at age 13 than girls, while parents observe lower self-control in adolescent sons. Bayesian research synthesis allowed us to compare and aggregate longitudinal results on the same concept measured with different instruments and by different cohorts, leading towards robust conclusions.

## Declaration of Competing Interest

None.
