# Supplementary material for: Robust longitudinal multi-cohort results: The development of self-control during adolescence
Source: Dev Cogn Neurosci. 2020 Jul 4;45:100817. doi: 10.1016/j.dcn.2020.100817 (PMC7451800; doi:10.1016/j.dcn.2020.100817)
Supplement: Supplementary file 1 [file mmc1.docx]

**Supplementary Materials**


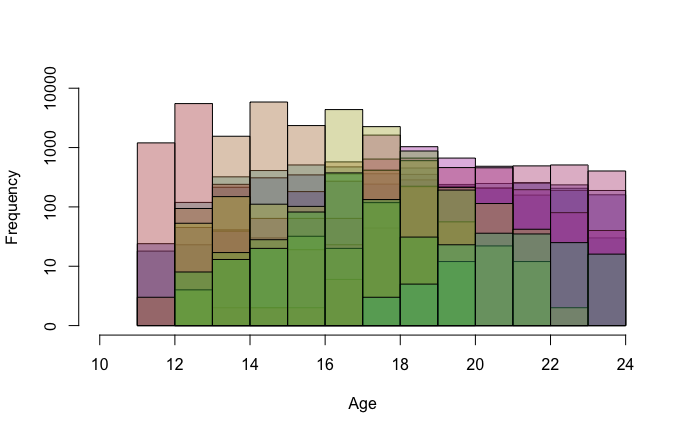


*Figure S1.* Distribution of age across 12 assessments for NTR with a log y-axis. *Note*: Wide age ranges for some assessments can be caused by the inclusion of younger and older siblings of the twins.


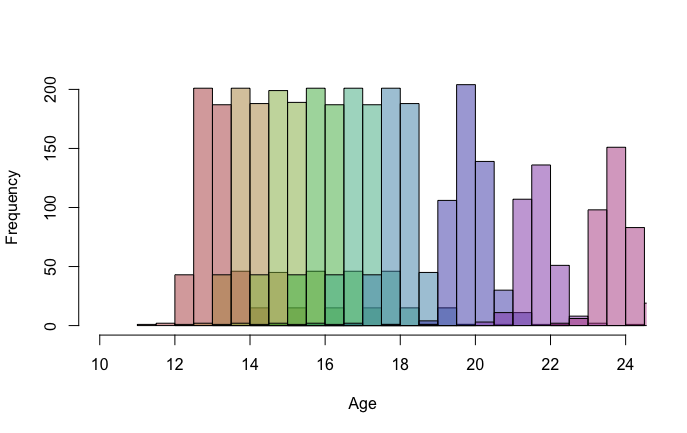


*Figure S2*. Distribution of age across Waves 1-9 in RADAR-Y.


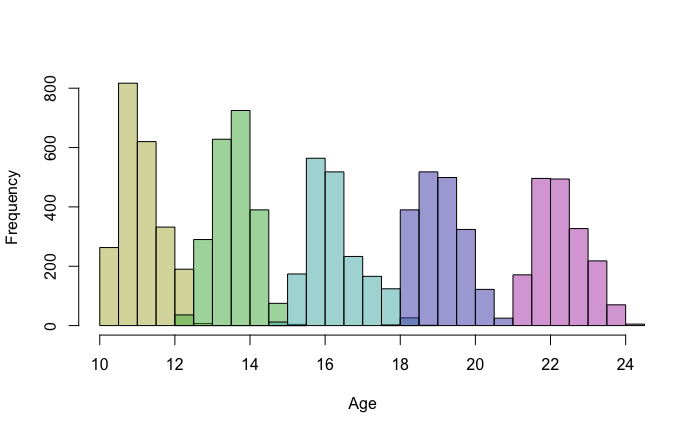


*Figure S3*. Distribution of age across Waves 1-5 in TRAILS.

|  | **H1** | **H2** | **H3** | **H4** | **H5** | **H6** | **Ha** |
| --- | --- | --- | --- | --- | --- | --- | --- |
| **NTR: ASCS** | .00 | .00 | .00 | .44 | .00 | .*56* | .00 |
| **RADAR-Y: ASCS-DERS** | .*81* | .12 | .03 | .03 | .00 | .00 | .07 |
| **TRAILS: ASCS** | .05 | .00 | .00 | *.82* | .03 | .10 | .00 |
| **TRAILS: Parent-ASCS** | 19 | .01 | .01 | *.72* | .03 | .04 | .00 |
| **TRAILS: EATQ** | .71 | .02 | .06 | .19 | .01 | .02 | .00 |
| **All** | .00 | .00 | .00 | **1.00** | .00 | .00 | .00 |

*Note.* Hypotheses: H1: ɑ_LS_ = 0 & σ_I,LS_ = 0, H2: ɑ_LS_ = 0 & σ_I,LS_ > 0, H3: ɑ_LS_ =0 & σ_I,LS_ < 0, H4: ɑ_LS_ < 0 & σ_I,LS_ = 0, H5: ɑ_LS_ < 0 & σ_I,LS_ > 0, H6: ɑ_LS_ < 0 & σ_I,LS_ < 0, Ha: ɑ_LS_ > 0, σ_I,LS_.

Table S1. *Posterior Model Probabilities for the hypotheses concerning self-control problem development and its covariance with initial self-control problem levels with the intercept centered at age 16.*

**S1. Missing Data Imputation**

Missing data were handled by means of multiple imputation with the R-package mice (multiple imputation by chained equations; Van Buuren & Groothuis-Oudshoorn, 2011). The item data were imputed in wide format, meaning that repeated assessments of one participant were included in one line in the data file (as opposed to long format where each time point is on a separate line). In this manner, measurements from previous and following occasions could easily be used to predict missing values, meanwhile preserving the dependency between repeated assessments for one person. Consequently, multilevel imputation methods were not required. In NTR, the wide data format did not only include repeated assessments, but also multiple persons from the same family.

For TRAILS and RADAR-Y, which both followed one cohort over time, missing data was imputed while the data was still structured by wave. In this way, we avoided creating data for non-existing assessments (e.g., a TRAILS participant who participated at age 14, never has an assessment at age 15 because the interval between waves is longer). NTR data, on the other hand, was restructured by age in years (i.e., the analysis format for the data) immediately, as none of the participants were invited to participate in all existing assessments and the overlapping assessment methods at age in years aided the imputation.

All datasets included repeated assessments of (1) self-control items for at least one questionnaire, (2) overall internalizing and externalizing problem scores, and (3) various items with background information. Given the large number of variables in the datasets, an initial predictor matrix for imputation was created based on minimum correlations of .25, and a minimum proportion of usable cases of .50. Repeated assessments of the same item, and assessments at the same age of a sibling were included as predictors in the imputation model. Sex was part of the analysis model, so it was included in the prediction model for all items and vice versa (see Van Buuren, 2018, Chapter 6). If the software indicated that variables were problematic predictors in the imputation model (e.g., because of collinearity), they were removed.

In general, the default imputation method was used, which is predictive mean matching for continuous variables and logistic regression imputation for binary categorical data (i.e., sex). Predictive mean matching samples values from other participants for the imputation, which means that it will never impute values that deviate from the measurement scale of interest.

Data were imputed 50 times. Imputation success was evaluated by checking the logged warnings and imputation traceplots. Imputation traceplots demonstrate imputation convergence if the lines over the iterations of each imputation have a stable mean and variance.
